# Serial change of C1 inhibitor in patients with sepsis: a prospective observational study

**DOI:** 10.1186/s40560-018-0309-5

**Published:** 2018-07-04

**Authors:** Tomoya Hirose, Hiroshi Ogura, Hiroki Takahashi, Masahiro Ojima, Kang Jinkoo, Youhei Nakamura, Takashi Kojima, Takeshi Shimazu

**Affiliations:** Department of Traumatology and Acute Critical Medicine, Osaka University Graduate School of Medicine, 2-15 Yamadaoka, Suita, Osaka 565-0871 Japan

**Keywords:** C1 inhibitor (C1-INH), Sepsis, Vascular permeability, Shock

## Abstract

**Background:**

C1 inhibitor (C1-INH), which belongs to the superfamily of serine protease inhibitors, regulates the complement system and also the plasma kallikrein-kinin, fibrinolytic, and coagulation systems. The biologic activities of C1-INH can be divided into the regulation of vascular permeability and anti-inflammatory functions. The objective of this study was to clarify the serial change of C1-INH in patients with sepsis and evaluate the relationship with the shock severity.

**Methods:**

This was a single-center, prospective, observational study. We serially examined C1-INH activity values (normal range 70–130%) in patients with sepsis admitted into the intensive care unit of the Trauma and Acute Critical Care Center at Osaka University Hospital (Osaka, Japan) during the period between January 2014 and August 2015. We defined “refractory shock” as septic shock unresponsive to conventional therapy such as adequate fluid resuscitation and vasopressor therapy to maintain hemodynamics.

**Results:**

Serial changes of C1-INH were evaluated in 40 patients with sepsis (30 men, 10 women; 30 survivors, 10 non-survivors; mean age, 70 ± 13.5 years). We divided the patients into three groups: non-shock group (*n* = 14), non-refractory shock group (*n* = 13), and refractory shock group (*n* = 13: 3 survivors, 10 non-survivors). In the non-shock group, C1-INH was 107.3 ± 26.5% on admission and 104.2 ± 22.3% on day 1, and it increased thereafter to 128.1 ± 26.4% on day 3, 138.3 ± 21.2% on day 7, and 140.3 ± 12.5% on day 14 (*p* < 0.0001). In the non-refractory shock group, C1-INH was 113.9 ± 19.2% on admission, 120.2 ± 23.0% on day 1, 135.7 ± 19.9% on day 3, 138.8 ± 17.2% on day 7, and 137.7 ± 10.7% on day 14 (*p* < 0.0001). In the refractory shock group, C1-INH was 96.7 ± 15.9% on admission, 88.9 ± 22.3% on day 1, 119.8 ± 39.6% on day 3, 144.4 ± 21.1% on day 7, and 140.5 ± 24.5% on day 14 (*p* < 0.0001). The difference between these three groups was statistically significant (*p* < 0.0001). C1-INH in non-survivors did not increase significantly during their clinical course (*p* = 0.0690).

**Conclusions:**

In refractory shock patients with sepsis, the values of C1-INH activity were lower (especially in non-survivors) on admission and day 1 as compared with non-shock and non-refractory shock patients.

## Background

C1 inhibitor (C1-INH), which belongs to the superfamily of serine protease inhibitors, regulates not only the complement system but also the plasma kallikrein-kinin, fibrinolytic, and coagulation systems [1, 2].The biologic activities of C1-INH can be divided into the regulation of vascular permeability and anti-inflammatory functions [1]. Hereditary angioedema (HAE), caused by an inherited deficiency of C1-INH, has been a focus in recent years [3]. During attacks of HAE, vascular permeability increases markedly, which leads to angioedema [4, 5].

The detailed pathology underlying increased vascular hyperpermeability in patients with HAE is not completely understood. Bradykinin is the main mediator of increased vascular permeability in patients with HAE [6] [7] During acute attacks of HAE, kallikrein is insufficiently inhibited because of the deficiency in C1-INH, the kallikrein-kinin system becomes activated, and at the end of the cascade, an increased amount of bradykinin is produced that results in the edema seen in patients with HAE.

In sepsis, significant vascular hyperpermeability is similarly observed systemically; however, the mechanism of vascular hyperpermeability in sepsis has not been completely elucidated [8, 9]. Cytokines and other inflammatory mediators induce gaps between endothelial cells by disassembly of intercellular junctions, by altering the cellular cytoskeletal structure, or by directly damaging the cell monolayer, and this creation of gaps can result in microvascular leakage and tissue edema, which are characteristic of sepsis [10]. Endothelial hyperpermeability is the key in the progression from sepsis to organ failure [9], but the role of C1-INH in the pathogenesis has not been clarified. In 1985, Kalter et al. [11] reported that C1-INH levels are significantly increased in uncomplicated bacteremia, moderately increased in patients with nonfatal episodes of bacterial shock, and not increased in those with fatal episodes. However, they did not evaluate the serial change of C1-INH levels, and the timing of blood sampling was unclear. Recently, in a preliminary report, we noted that C1-INH activity was not enhanced in two refractory shock patients with sepsis (one survivor and one non-survivor) on admission to hospital. The surviving patient’s general condition had improved with increases in C1-INH activity, and enhancement of C1-INH activity was also observed in three non-refractory shock patients with sepsis [12, 13].

Thus, the objectives of this study were to prospectively evaluate the serial changes of C1-INH activity in a larger population of patients with sepsis under the current standard treatment policy and to evaluate the relationship with the shock severity.

## Methods

### Patients and setting

This was a single-center, prospective, observational study that was approved by the Ethics Committee of Osaka University Graduate School of Medicine. From January 2014 to August 2015, we examined blood samples collected from patients with sepsis admitted into the intensive care unit of the Trauma and Acute Critical Care Center at Osaka University Hospital (Osaka, Japan). Sepsis and septic shock were diagnosed according to the “Surviving Sepsis Campaign: International Guidelines for Management of Severe Sepsis and Septic Shock: 2012” [14]. Exclusion criteria were age < 15 year and end stage of malignant disease.

Our principle therapeutic policy regarding circulation management for sepsis is as follows. Initial resuscitation is performed according to the “Surviving Sepsis Campaign: International Guidelines for Management of Severe Sepsis and Septic Shock: 2012” [14]. Even with adequate fluid resuscitation and vasopressor therapy (noradrenalin of > 0.1 μg/kg/min div. for more than at least 1 h), if the arterial systolic pressure is < 90 mmHg, we administer intravenous hydrocortisone (initial dose 100-mg bolus intravenously and then 200 mg per day via continuous intravenous administration).

We defined “refractory shock” as septic shock unresponsive to conventional therapy such as adequate fluid resuscitation and vasopressor therapy to maintain hemodynamics. We divided the patients into three groups: the non-shock group, the non-refractory shock group, and the refractory shock group. We obtained all necessary consents from all patients and their kin involved in this study.

### Evaluation of clinical background and C1-INH activity

The patients’ clinical background and course including age, sex, Acute Physiological and Chronic Health Evaluation (APACHE) II score, Sequential Organ Failure Assessment (SOFA) score, prognosis, infusion volume, catecholamine administration, and steroid administration were recorded. We serially examined C1-INH activity values (normal range 70–130%) in patients with sepsis. The timing of sampling was day 0 (at admission), 1, 3, 5, 7, 10, and 14. The blood samples were stored at − 80°C until C1-INH activity values were measured in plasma samples using a Berichrom C1-INHibitor kit (Siemens Healthcare Diagnostics, Deerfield, IL) according to the manufacturer’s instructions.

### Statistical analysis

All data are presented as the mean ± standard deviation (SD) except that in the figure captions, which are the mean ± standard error of the mean (SEM). To compare the baseline characteristics of the subjects in the three groups, analysis of variance (ANOVA) was used for the continuous values. Differences in longitudinal data between the groups were tested by repeated measures ANOVA. A *p* value > 0.05 was considered to indicate statistical significance. All statistical analyses were performed using JMP Pro 11.2.0 (SAS Institute Inc., Cary, NC).

## Results

### Patient characteristics

The serial changes of C1-INH activity values were evaluated in 40 patients with sepsis (30 men and 10 women; 30 survivors and 10 non-survivors; mean age, 70.0 ± 13.5 years): the non-shock group (*n* = 14), the non-refractory shock group (*n* = 13), and the refractory shock group (*n* = 13: 3 survivors, 10 non-survivors). The characteristics of these groups are shown in Table 1. Among the three groups, the volume of infusion required during the first 48 h after admission to maintain hemodynamics was the greatest in the refractory shock group. The relationship between infusion volume required during the first 48 h and C1-INH activity at day 0 was not statistically significant (*p* = 0.1104).

**Table 1:** Patient characteristics

Characteristic	Non-shock	Non-refractory shock	Refractory shock	*p*
*N*	14	13	13	
Age (± SD) (years)	66.8 ± 17.1	72.5 ± 10.4	71.0 ± 12.2	0.5346
Male (%)	11 (78.6)	9 (69.2)	10 (76.9)	0.8388
Survivor *n* (%)	14 (100)	13 (100)	3 (23.1)	< .0001
APACHE II score	13.1 ± 5.9	25.5 ± 5.7	27.9 ± 10.2	< .0001
SOFA score	4.3 ± 0.9	9.4 ± 0.9	9.7 ± 0.9	0.0001
Volume of infusion over the first 48 h after admission (± SD) (mL)	9735.5 ± 6852.9	11,765.2 ± 6369.4	18,390.7 ± 8908.8	0.0127
Mean volume of infusion/h over the first 48 h after admission (± SD) (mL/h)	204.3 ± 140.6	263.9 ± 146.2	484.0 ± 146.3	< .0001
Diagnosis (*n*)				
Pneumonia	1	3	3	
Urinary tract infection	2	2	3	
Gas gangrene	3	1	2	
Abdominal infection	3	5	2	
CNS infection	1	1	0	
Infective endocarditis	1	0	0	
Cellulitis	1	1	3	
Others	2	0	0	

*APACHE* Acute Physiological and Chronic Health Evaluation, *CNS* central nervous system, *SD* standard deviation, *SOFA* Sequential Organ Failure Assessment

### Comparison of serial changes of C1-INH activity between groups

**Fig. 1:**
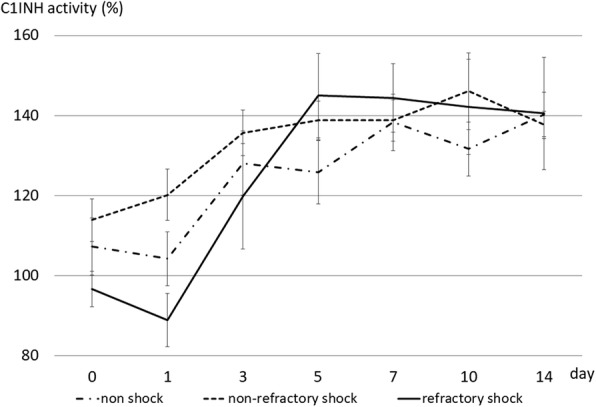
Comparison of the serial changes of C1-INH activity between the three groups. In the non-shock group, C1-INH was 107.3 ± 26.5% on admission and 104.2 ± 22.3% on day 1, and it increased to 128.1 ± 26.4% on day 3, 138.3 ± 21.2% on day 7, and 140.3 ± 12.5% on day 14) (*p* < 0.0001). In the non-refractory shock group, C1-INH was 113.9 ± 19.2% on admission, and it increased thereafter to 120.2 ± 23.0% on day 1, 135.7 ± 19.9% on day 3, 138.8 ± 17.2% on day 7, and 137.7 ± 10.7% on day 14) (*p* < 0.0001). In the refractory shock group, C1-INH was 96.7 ± 15.9% on admission, it dropped to 88.9 ± 22.3% on day 1, and then it increased to 119.8 ± 39.6% on day 3, 144.4 ± 21.1% on day 7, and 140.5 ± 24.5% on day 14) (*p* < 0.0001). The difference between these three groups was statistically significant (*p* < 0.0001). The normal range of C1-INH activity values is 70–130%

A comparison of the serial changes of C1-INH activity values between the three groups is shown in Fig. 1. In the non-shock group, C1-INH was 107.3 ± 26.5% on admission and 104.2 ± 22.3% on day 1. Thereafter, it increased to 128.1 ± 26.4% on day 3, 138.3 ± 21.2% on day 7, and 140.3 ± 12.5% on day 14 (*p* < 0.0001). In the non-refractory shock group, C1-INH was 113.9 ± 19.2% on admission, and it increased thereafter to 120.2 ± 23.0% on day 1, 135.7 ± 19.9% on day 3, 138.8 ± 17.2% on day 7, and 137.7 ± 10.7% on day 14 (*p* < 0.0001). In the refractory shock group, C1-INH was 96.7 ± 15.9% on admission, it dropped to 88.9 ± 22.3% on day 1, and then increased to 119.8 ± 39.6% on day 3, 144.4 ± 21.1% on day 7, and 140.5 ± 24.5% on day 14 (*p* < 0.0001). The difference between these three groups was statistically significant (*p* < 0.0001).

### Serial change of C1-INH activity in each patient in the refractory shock group

**Fig. 2:**
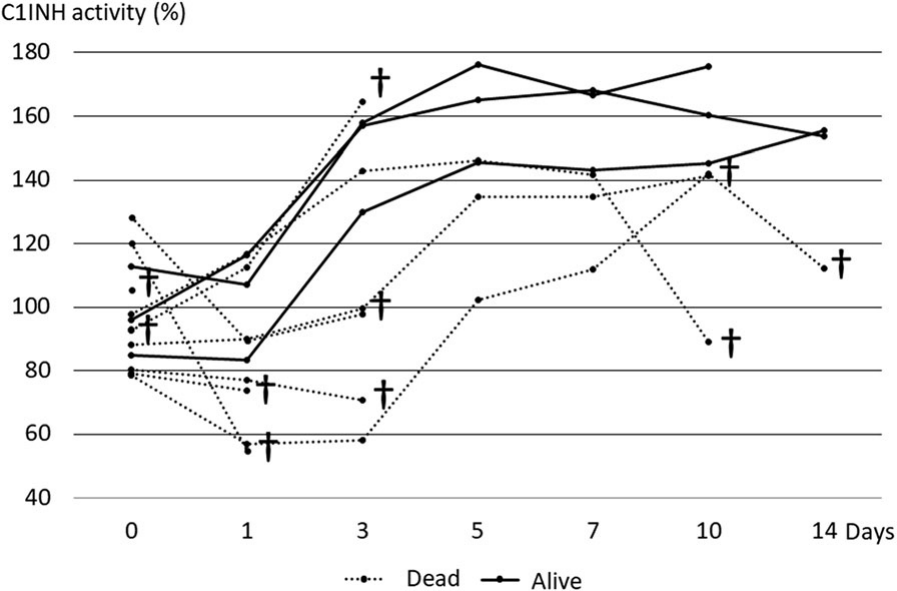
Serial changes of C1-INH activity in the 13 patients in the refractory shock group (3 survivors, 10 non-survivors). C1-INH activity increased in all survivors after admission, but in the non-survivors, it did not necessarily increase after admission. ^†^Dead. The normal range of C1-INH activity values is 70–130%

Serial changes of C1-INH activity values in each patient in the refractory shock group are shown in Fig. 2. C1-INH activity increased after admission in the three survivors, but it did not necessarily increase after admission in the non-survivors. Some patients died because hemodynamics could not be maintained during the first few days after admission, and others died because of multiple organ failure at more than 1 week after admission.

### Comparison of serial changes of C1-INH activity between survivors and non-survivors in the refractory shock group

**Fig. 3:**
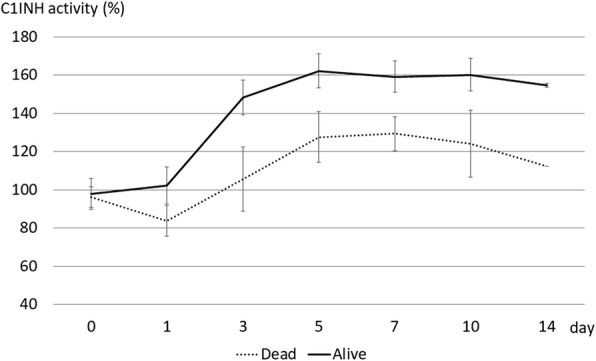
Comparison of overall serial changes of C1-INH activity between the survivors and non-survivors in the refractory shock group. During the clinical course, C1-INH increased significantly in the survivors (*p* < 0.0001) but did not increase significantly in the non-survivors (*p* = 0.0690). The difference between these two groups was statistically significant (*p* < 0.0001). The normal range of C1-INH activity values is 70–130%

Serial changes of C1-INH activity between the survivors and non-survivors in the refractory shock group are compared in Fig. 3. Over the clinical courses, C1-INH increased significantly in the survivors (*p* < 0.0001) but did not increase significantly in the non-survivors (*p* = 0.0690).

## Discussion

In this study, we showed the serial changes of C1-INH activity values in patients with sepsis. Septic patients are reported to often exhibit a relative deficiency of C1-INH [15, 16]. The findings in our previous preliminary study suggested that C1-INH activity may be suppressed in patients with refractory shock due to the enhanced consumption or suppressed production of C1-INH [12, 13].

In sepsis, significant endothelial hyperpermeability similar to that of HAE is observed systemically [8]. In the present study, the highest values of C1-INH activity were found in the non-refractory shock group, followed by those in the non-shock group and those in the refractory shock group, especially on days 0 and 1 (Fig. 1). We thought that C1-INH works to suppress vascular permeability; thus, the C1-INH activity values in the non-refractory shock group increased, whereas those in the refractory shock group decreased due to the enhanced consumption or suppressed production of C1-INH. As a result, the patients in the refractory shock group required a high volume of fluid resuscitation (Table 1), and these patients might develop a relative deficiency of C1-INH. It is presently not clear how high the C1-INH activity value should be throughout sepsis treatment, especially in refractory shock patients. Further study is required to evaluate this point.

Animal studies showed that C1-INH administration improves vascular permeability [17, 18]. Schmidt et al. [17] revealed that pretreatment with C1-INH attenuates macromolecular leakage in postcapillary venules of rat mesentery, and Liu et al. [18] reported that C1-INH suppresses the systemic lipopolysaccharide-induced increase in microvascular permeability in mice. In CLP (cecal ligation and puncture) models of sepsis, treatment with a single dose of C1-INH improved survival as reported by Liu et al. [19]. Some validity for the administration of C1-INH in the treatment of sepsis has been shown by animal models such as these [20].

In contrast, there is very little clinical data on C1-INH administration in patients with sepsis [20]. Recently, Igonin et al. [21] reported that C1-INH infusion increased survival rates for patients with sepsis in an open-label, randomized, controlled study. C1-INH administration in patients with sepsis was associated with reduced all-cause mortality (12 vs. 45% in the control, *p* = 0.008) and sepsis-related mortality (8 vs. 45% in the control, *p* = 0.001) assessed over 28 days. However, their study population was small (C1-INH group: *n* = 42, control group: *n* = 20), one of their inclusion criteria was that patients begin treatment within 48 h of sepsis onset, and C1-INH activity values were not evaluated. We thought that the inclusion criteria of their study on C1-INH replacement therapy in patients with sepsis may have been focused on refractory shock cases. Further study is required to evaluate the effect of C1-INH replacement therapy in sepsis.

In sepsis, the complement system including C1-INH has an important role in the host defense against bacterial infection, and activation of the complement system through the classic, alternative, and lectin pathways leads to inflammatory host response [15, 20]. These pathways include various component factors such as C1, C2, C3, C4, C5, C6, C7, C8, C9, MBL (mannose-binding lectin), and MASP2 (MBL-associated serine proteases) [20]. C1-INH regulates the complement system such as C1r, C1s, and MASP2 [1]. We only evaluated C1-INH activity in the present study. Therefore, the evaluation of serial changes in the other complemental factors is also needed to clarify the role of C1-INH regulation of hyperpermeability in human sepsis patients.

Our study has some limitations. First, we only evaluated C1-INH over the first 2 weeks after admission, and the long-term change in C1-INH was not clarified. Second, complement factors other than C1-INH were not evaluated. Third, we only evaluated fluid volume and did not evaluate markers of vascular endothelial dysfunction such as glycocalyx injury or changes in bradykinin concentration. Fourth, we may have to consider the effect of dilution by fluid infusion. Fifth, the sample size was small. Finally, we only evaluated C1-INH activity, not C1-INH quantitative values, in the present study. In our previous study, C1-INH quantitative values were low on admission in refractory shock patients even though they had normal C1-INH activity values [12, 13]. Because we have no data on C1-INH quantitative values in the present study, further evaluation is required on this point.

Further prospective, randomized, control studies to validate C1-INH replacement therapy including the evaluation of C1-INH and other complement factors in a larger population with sepsis are needed.

## Conclusions

In refractory shock patients with sepsis, the values of C1-INH activity were lower (especially in non-survivors) on admission and day 1 as compared with non-shock and non-refractory shock patients.

## Abbreviations

APACHE: Acute Physiological and Chronic Health Evaluation
C1-INH: C1 inhibitor
HAE: Hereditary angioedema
SD: Standard deviation
SEM: Standard error of the mean
